# Flightless I Negatively Regulates Macrophage Surface TLR4, Delays Early Inflammation, and Impedes Wound Healing

**DOI:** 10.3390/cells11142192

**Published:** 2022-07-13

**Authors:** Stuart J. Mills, Parinaz Ahangar, Hannah M. Thomas, Benjamin R. Hofma, Rachael Z. Murray, Allison J. Cowin

**Affiliations:** 1 Regenerative Medicine, Future Industries Institute, University of South Australia, Mawson Lakes, Adelaide SA 5095, Australia; parinaz.ahangar@unisa.edu.au (P.A.); hannah.mary.milroy.thomas@gmail.com (H.M.T.); ben.hofma@hri.org.au (B.R.H.); 2 School of Biomedical Sciences, Faculty of Health, Queensland University of Technology, Brisbane QLD 4059, Australia; rachael.murray@qut.edu.au

**Keywords:** Flightless I, inflammation, macrophage, toll-like receptor (TLR4), skin, wound healing

## Abstract

TLR4 plays a pivotal role in orchestrating inflammation and tissue repair. Its expression has finally been balanced to initiate the early, robust immune response necessary for efficient repair without excessively amplifying and prolonging inflammation, which impairs healing. Studies show Flightless I (Flii) is an immunomodulator that negatively regulates macrophage TLR4 signalling. Using macrophages from Flii^+/−^, WT, and Flii^Tg/Tg^ mice, we have shown that elevated Flii reduces early TLR4 surface expression, delaying and reducing subsequent TNF secretions. In contrast, reduced Flii increases surface TLR4, leading to an earlier robust TNF peak. In Flii^+/−^ mice, TLR4 levels peak earlier during wound repair, and overall healing is accelerated. Fewer neutrophils, monocytes and macrophages are recruited to Flii^+/−^ wounds, leading to fewer TNF-positive macrophages, alongside an early peak and a robust shift to M2 anti-inflammatory, reparative Ym1^+^ and IL-10^+^ macrophages. Importantly, in diabetic mice, high Flii levels are found in plasma and unwounded skin, with further increases observed in their wounds, which have impaired healing. Lowering Flii in diabetic mice results in an earlier shift to M2 macrophages and improved healing. Overall, this suggests Flii regulation of TLR4 reduces early inflammation and decreases the M2 macrophage phenotype, leading to impaired healing.

## 1. Introduction

The consequences of skin injury are predictable for most individuals. Once blood loss is stemmed, an inflammatory phase, characterised by the infiltration of neutrophils and monocyte/macrophages, instigates the complex process of restoring skin integrity [1]. New tissue is formed to cover the injury, and wound maturation commences. The role of the immune system in the repair process is complex [1]. An initial, robust burst of inflammation is essential for successful wound repair; however, prolonged inflammation has been shown to be detrimental to healing outcomes, as is the case for many chronic wounds, such as diabetic ulcers [2].

Toll-like receptors (TLRs) play a pivotal role in this early inflammatory burst. Upregulated during wound healing, they bind to the pathogen-associated molecular patterns (PAMPs) and damage-associated molecular patterns (DAMPs) that are released from injured and necrotic cells, as well as damaged extracellular ECM components, driving the inflammatory response [3]. The binding of DAMPs and PAMPs to TLR4 initiates signalling cascades through the recruitment of the adaptor protein MyD88 to the cytoplasmic tail of TLR4, culminating in the activation of transcription factors, such as NF-κB, that control the expression of an array of cytokines, for example TNF, that dictate the outcome of innate immune responses. The temporal increase in TLR4 expression seen after injury is necessary for timely repair, and mice deficient in TLR4 or MyD88 show delayed healing [4,5,6]. Adoptive transfer experiments show that the transfer of wildtype monocytes/macrophages rescues the delayed wound healing in TLR4-deficient mice, suggesting that the TLR4 on monocytes/macrophages is a key source of TLR4 during wound repair [4].

TLR4 also plays a role in macrophage polarisation [7,8]. Macrophages polarise into two broad phenotypes, M1 or M2, although this is an oversimplification as these are not discrete populations, but rather represent a continuum of phenotypes [9,10]. The timing of the transition to the M2 phenotype dictates the duration and severity of an inflammatory response. This switch from M1 to M2 reflects a change in function, from inflammatory to reparative, and wounds that have impaired healing tend to have more M1 macrophages and fewer M2 macrophages [1,11]. Data suggest that the upregulation of TLR4 on macrophages might drive macrophages towards an M1 proinflammatory phenotype, and the deletion of myeloid TLR4 has been shown to promote their differentiation into an anti-inflammatory, reparatory M2-like phenotype [7,8].

Flightless I (Flii) is an immunomodulatory protein that regulates TLR4 signalling and has been shown to alter wound healing, with high levels delaying wound closure [12]. A number of cell types found in the skin express Flii, including macrophages [13,14]. Both TLR4 and Flii contain leucine-rich-repeat (LRR) domains which, in the case of TLR4, have been shown to bind to specific DAMPs and PAMPs, including LPS [15]. Flii has been found to also bind to LPS and potentially binds to other PAMPs and DAMPs [16]. Flii is located in the cytosol and can be secreted, where it has been detected in wound fluid and plasma [16]. The extracellular pool of Flii can prevent LPS binding to TLR4 on macrophages and dampen inflammation [16]. Inside the cell, Flii can bind MyD88, interfering with the formation of the TLR4-MyD88 signalling complex, and it also inhibits NLRP3 inflammasome activation [15,16,17,18,19,20,21].

We have previously shown Flii to be upregulated in response to wounding and to negatively regulate the healing response; however, the mechanism underpinning Flii activity during inflammation and wound repair is still not fully understood. In this study, we show that Flii negatively regulates TLR4 and alters both the level and timing of TNF secretion. In wounds, this prolongs inflammation with a delay in the switch to the M2 reparative macrophage phenotype, leading to impaired healing. Reduced levels of Flii lead to shorter, reduced inflammatory responses with earlier switches to M2 macrophages and enhanced wound closure. In a mouse diabetic model, increased Flii levels also delayed healing, with increased inflammation and a delayed switch to the M2 macrophage phenotype.

## 2. Materials and Methods

### 2.1. Antibodies and General Reagents

Primary antibodies used for the immunohistochemistry are as follows: TLR4 (14-9917-82) and Flightless I (PA5-21735) were purchased from Thermo Fisher Scientific (Waltham, MA, USA). Neutrophil marker NIMP-R14 (sc-59338) and TNF (sc-52746) were purchased from Santa Cruz (Dallas, TX, USA); IL-10 (ab189392) were purchased from Abcam (Melbourne, Australia); Ly-6C (551459) were purchased from BD Biosciences (New South Wales, Australia); F4/80 (MCA4975) were purchased from BioRad (New South Wales, Australia); and Ym1 (60130) were purchased from StemCell (StemCell Technologies, Tullamarine, Australia). Secondary antibodies—goat anti-rabbit Alexa Fluor 635, donkey anti-rabbit Alexa Fluor 568, donkey anti-goat Alexa Fluor 633, and donkey anti-rat Alexa Fluor 488—were purchased from Thermo Fisher Scientific (USA). For Western blotting, Flii (sc-30046) were purchased from Santa Cruz Biotechnologies (Dallas, TX, USA), and anti-rabbit antibodies (#P0448) were purchased from Agilent (Santa Clara, CA, USA). For flow cytometry (FACs), TLR4 (564215) and F4/80 (567201) were purchased from BD Biosciences (Australia).

### 2.2. Murine Models of Wound Healing

All experiments were approved by the Adelaide Women’s and Children’s Health Network Animal Ethics Committee and the University of South Australia Animal Ethics Committee, and they followed the Australian Code for the Care and Use of Animals for Scientific Purposes (AE1021/10/18). Upon arrival from the Animal Resources Centre (ARC), mice were group-housed, allowed to acclimatise for 7 days, and kept in a 12-h day/night cycle.

All mice were raised on a BALB/C background, and the wildtype mice were designated as WT. Mice with a heterozygous knockout retained one copy of the Flii gene and were designated Flii^+/−^. Flii-overexpressing mice were homozygous for a human Flii transgene in addition to the two endogenous copies of the murine Flii gene and were designated Flii^Tg/Tg^. The generation of these strains has been described previously [22].

Diabetes was induced in 12-week-old female Flii^+/−^, WT, and Flii^Tg/Tg^ mice (*n* = 8), with body weights of 18–22 g, by streptozotocin (Sigma-Aldrich, Darmstadt, Germany) intraperitoneal injection, as described previously [23]. Diabetes was confirmed by the verification of a consistently elevated (>15.25 mg/L) blood glucose level (BGL) for a sustained period of >6 weeks.

On the day of surgery, mice were anaesthetised using 2 L/min of isoflurane and oxygen inhalation and administered analgesics. A total of 2 full-thickness, 1-centimetre incisions were made on the dorsum of the Flii^+/−^, WT, and Flii^Tg/Tg^ mice. The incisions were 1 cm from the occipital protuberance and 1 cm from either side of the midline. The mice were then individually caged, and the wounds were left to heal by secondary intention until the endpoint of day 1, 2, 3, 5, or 7.

All wounds were photographed daily for macroscopic measurements. At the endpoints, wounds were removed and fixed in 10% neutral-buffered formalin for 24 h and then processed into paraffin for immunohistochemistry.

### 2.3. Immunohistochemistry

Mouse sections were cut from the paraffin-embedded tissue at 4 µm using a microtome. Antigen retrieval was performed by heating the sections, using a pressure cooker, in 0.1 M citric acid buffer (pH 7.4) at 90 °C for 10 min and then allowed to cool to room temperature. All antibodies were used at 1:100 dilution, and appropriate secondary antibodies were used at 1:200 dilution. a total of 5 drops of diamidino-2-phenylindole (DAPI; 1:10,000) were added to each section and incubated for 5 min prior to the end of the staining procedure.

### 2.4. Macrophage Collection

Macrophages were collected from the peritoneum of the three Flii mouse genotypes. Mice were humanely killed via CO_2_ overdose, and 10 ml of ice-cold sterile PBS was injected into the peritoneum and then removed using a syringe. The cells were centrifuged and resuspended in RPMI with 10% foetal calf serum (Merck, Bayswater, Australia) and incubated for 24 h at 37 °C and 5% CO_2_. Macrophages were treated with or without 5 µg LPS (Merck, Australia) to activate them. Media or cells were collected for ELISA or flow cytometry at 3, 6, 12, 24, 48, and 72 h.

### 2.5. Cytokine ELISA

A mouse TNF ELISA (In Vitro Technologies, Noble Park North, Australia) kit was used per the manufacturer’s instructions to measure TNF levels in the media from the in vitro macrophage experiments.

### 2.6. Flow Analysis (FACs)

**Figure 1:**
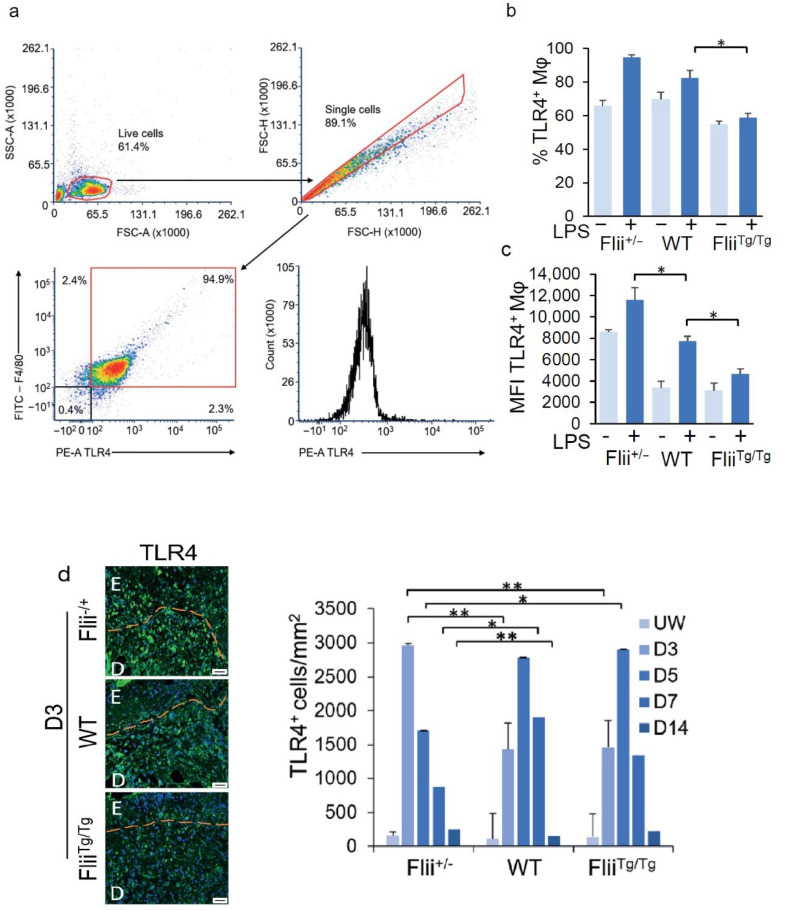
TLR4 expression levels in murine macrophages and wounds from three Flii genotypes (Flii^+/−^, WT, and Flii^Tg/Tg^): (**a**) Representative gating strategy for macrophage TLR4 expression; Flow cytometry analysis showing (**b**) percentage and (**c**) mean fluorescent intensity of TLR4^+^ murine macrophages from the 3 Flii genotypes, with and without LPS stimulation (*n* = 6); (**d**) Representative images and bar graph showing the number of TLR4^+^ cells within acute murine wounds of the three Flii genotypes after 3, 5, 7, and 14 days (*n* = 6). Scale bars = 50 µm. Results are expressed as mean ± SEM. * *p* < 0.05 and ** *p* < 0.05.

Cells were washed in PBS before being stained with Fixable Viability Stain 780 (BD Biosciences, Australia), diluted 1/1000, for 10 min. Cells were then washed in FACS buffer (PBS 1% BSA, 0.04% azide) and stained for 30 min with directly conjugated antibodies to TLR4 (1:200) and F4/80 (1:200) in Brilliant Stain Buffer (566349; BD Biosciences, Australia). After staining, cells were washed once with FACS buffer and once with PBS. Stained cells were acquired on the BD LSRFortessa X-20 flow cytometer and data analysis was performed using Diva Software (BD Biosciences). Gating strategies are detailed in Figure 1a.

### 2.7. Western Blotting

Western blot analysis of Flii protein expression was carried out on mouse plasma from healthy and diabetic mice, as previously described [22]. SDS-PAGE loading buffer (3 μL of 25 nM Tris, pH 6.8; 8% glycerol; 1% SDS; 0.02% bromophenol blue; and 5% 2-mercaptoethanol) was added to 15 μL of each protein sample. Samples were boiled at 100 °C for 5 min and then vortexed. Samples (15 μL) were separated using a 10% polyacrylamide gel (sodium dodecyl sulphate-polyacrylamide gel electrophoresis (SDS-PAGE)). Electrophoresis was performed in 1× Western Running Buffer (25 mM Tris, glycine, 192 mM, 0.1% SDS) at 25 mA per gel, and transfer was carried out at 250 mA for 1 h at 4 °C. The membrane was blocked in 15% skim milk in PBS for 1 h and blotted with primary antibodies against Flii (1:200) in 6% skim milk overnight at 4 °C. The membrane was washed 3 times for 5 min in 1× TBST (TBS, 0.1% Tween20) and probed with secondary antibodies (1:2000) for 1 h at room temperature. The membrane was washed 2 times, developed using a Clarity Western ECL kit (Bio-Rad, Gladesville, NSW, Australia), and imaged under UV light using a SynGene G-Box. Results were representative of three independent experiments.

### 2.8. Statistical Analysis

The analysis of the wound measurements was carried out using Image ProPlus software V7 (MediaCybernetics Inc., Rockville, MD, USA; https://www.meyerinst.com/mediacybernetics/image-pro-plus/, accessed on 12 January 2022). Cell counts and intensity measurements were carried out using CellSens software V1.18 (Olympus, Australia; https://www.olympus-lifescience.com/en/software/cellsens, accessed on 29 June 2022). Statistical differences were determined using ANOVA, with the Bonferroni correction for multiple groups and Student’s *t*-test. For non-parametric data, a Mann–Whitney *U* test was used. A *p* value of <0.05 was considered significant.

## 3. Results

### 3.1. Increased Flii Levels Reduce Macrophage Surface TLR4

Activation of the TLR4 signalling pathway increases TLR4 surface expression in macrophages, and Flii negatively regulates this TLR4 signalling pathway [2,4,16]. To determine whether altering Flii levels modifies TLR4 surface expression on macrophages, mouse peritoneal macrophages were obtained from 3 Flii genotypes, Flightless heterozygous (Flii^+/−^, 50% levels of Flii), wildtype BALB/C (WT, 100% levels of Flii), and Flightless transgenic (Flii^Tg/Tg^, 150% levels of Flii) [24]. The macrophages were stimulated with or without LPS for 24 h and examined for TLR4 surface receptors by flow cytometry (Figure 1a–c). Between 55–70% of the inactivated macrophages were TLR4^+^, and LPS activation led to an increase in TLR4^+^ macrophages across the 3 genotypes (Figure 1b). However, as Flii levels increased, this increase in TLR4^+^ macrophages was significantly lesser; macrophages with low levels of Flii (Flii^+/−^) increased from 66% to 95%, WT macrophages from 70% to 82%, and those expressing high levels of Flii (Flii^Tg/Tg^) only shifted from 55% to 58% after LPS activation (Figure 1b). Surface levels (mean fluorescence intensity; MFI) were also significantly altered by changes in the level of Flii (Figure 1c). As the level of Flii decreases, the level of surface TLR4 increases, with a 1.6-fold increase in WT and a 2.5-fold increase in Flii^+/−^ as compared to the Flii^Tg/Tg^ LPS-activated macrophages (Figure 1c). Together, this data suggests that Flii has the ability to alter the level of surface TLR4 on macrophages.

### 3.2. Flii Decreases the Number of TLR4 Positive Cells in the Early Stages of Wound Healing

TLR4 is upregulated in response to injury [3,6]; therefore, incisional wounds were created on all 3 mouse genotypes to determine whether Flii might regulate TLR4 expression in vivo. The number of TLR4^+^ cells in the unwounded skin of all 3 genotypes was relatively low in the epidermis and dermis, but that number significantly increased after injury, returning to near-basal levels by day 14 post-injury, when the inflammatory and proliferative stages of wound repair were complete (Figure 1d). The number of TLR4^+^ cells peaked around day 3 in the wounds with low levels of Flii (Flii^+/−^), with a 19-fold increase compared to unwounded skin; these levels reduced to 5.7-fold that of unwounded tissue by day 5 (Figure 1d). In contrast, TLR4^+^ cells in WT and Flii^Tg/Tg^ wounds peaked later, at day 5, with a 22–23-fold increase compared to unwounded skin (Figure 1d). These data suggest that the level of Flii might regulate TLR4 expression in wounds, and that lower levels of Flii lead to an early peak in TLR4+ cells that resolves earlier in the healing process.

### 3.3. Flii Delays Inflammatory Cell Recruitment to the Wound

**Figure 2:**
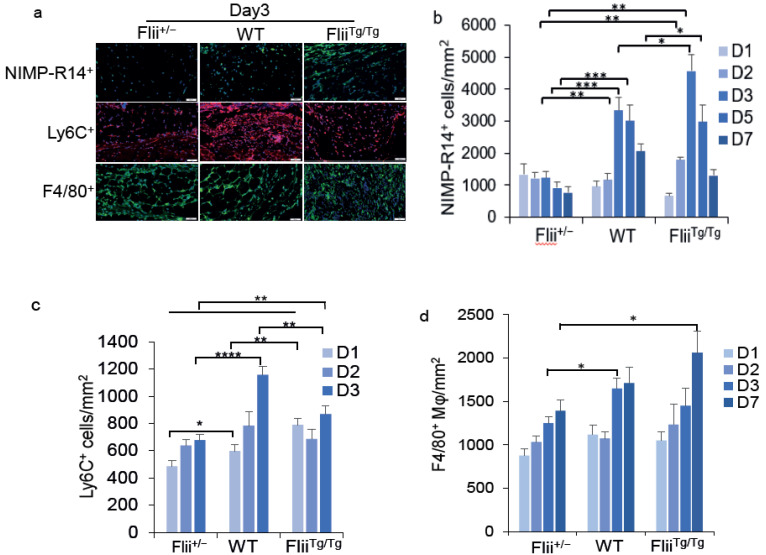
Wound neutrophils, monocytes, and macrophages for the Flii genotypes (Flii^+/−^, WT, and Flii^Tg/Tg^): (**a**) Representative images of neutrophil (NIMP-R14), monocyte (Ly6C), and macrophage (F4/80) staining in wounds from the 3 Flii genotypes at day 3; Bar graphs showing (**b**) neutrophil, (**c**) monocyte, and (**d**) macrophage cell numbers within the wounds of the 3 Flii genotypes (*n* = 6). Scale bars = 50 µm. Results are expressed as mean ± SEM. * *p* < 0.05, ** *p* < 0.05, *** *p* < 0.001 and **** *p* < 0.0001.

TLR4 signalling regulates the influx of immune cells to the wound [5]; therefore, the manner by which Flii affected the timing and number of immune cells entering the wound was next investigated. No significant difference in neutrophils (NIMP-R14^+^ cells) was observed in day-1 wounds between the 3 mouse genotypes, and for Flii^+/−^ wounds, the number of neutrophils did not increase over time, but instead decreased steadily over the 7-day timecourse (Figure 2a,b). In contrast, neutrophil numbers in the wounds of both WT and Flii^Tg/Tg^ mice significantly increased over time, peaking at day 3 at 2.7-fold and 3.7-fold, respectively, as compared to Flii^+/−^ wounds, and gradually reducing over 7 days (Figure 2a,b).

Monocytes (Ly6C^+^) were detected in the murine wounds from all 3 genotypes from day 1, with numbers increasing over time; however, there were significantly lower levels of monocytes recruited to the Flii^+/−^ mice’s wounds, as compared to the WT and Flii^Tg/Tg^ mice’s wounds (1.2-fold and 1.6-fold less, respectively) (Figure 2a,c). By day 3, the WT and Flii^Tg/Tg^ mice’s wounds had significantly more (1.7-fold and 1.3-fold) monocytes than the Flii^+/−^ mice’s wounds (Figure 2a,c). Monocytes in the wound mature into macrophages [25]. Over a 7-day timecourse, macrophage (F4/80^+^ cells) numbers increased in the wounds of all 3 mouse genotypes (Figure 2a,d). However, by day 7, it could be seen that, as the levels of Flii increased, so did the numbers of macrophages, with 1.2-fold and 1.5-fold more macrophages in the WT and Flii^Tg/Tg^ mice’s wounds (Figure 2a,d). Overall, these data suggest that the lower the level of Flii, the lower the number of neutrophils, monocytes, and macrophages infiltrating the wound.

### 3.4. Flii Inhibits Macrophage NF-κB Phosphorylation, TNF Secretion, and the Number of TNF^+^ Wound Macrophages in the Early Stages of Wounding

**Figure 3:**
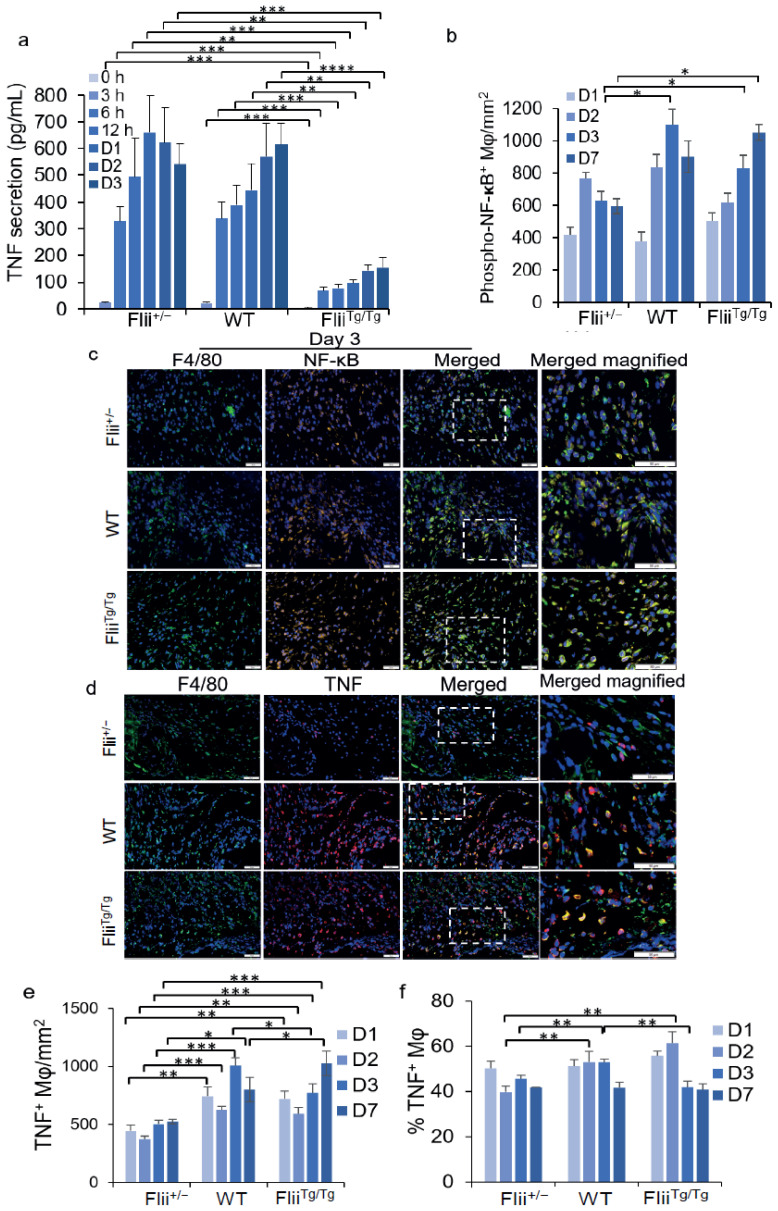
TNF secretion from macrophages and expression in wound macrophages from three Flii genotypes (Flii^+/^^−^, WT, and Flii^Tg/Tg^): (**a**) TNF secretion from LPS-activated macrophages; (**b**) representative images of murine wounds immunostained for TNF^+^ and F4/80^+^ at day 3; Bar charts showing (**c**) the number and (**d**) the percentage of TNF^+^ F4/80^+^ macrophages in the wounds of the 3 Flii genotypes; (**e**) Representative images of murine wounds immunostained for phosphorylated NF-κB^+^ and F4/80^+^ at day 3; (**f**) Bar chart showing the number of phosphorylated NF-κB^+^ F4/80^+^ macrophages in the wounds of the 3 Flii genotypes (*n* = 6). Scale bars = 50 µm. Results are expressed as mean ± SEM. * *p* < 0.05, ** *p* < 0.01, *** *p* < 0.001, and **** *p* < 0.0001.

Flii can alter macrophage TLR4 levels (Figure 1b,c). Signalling through the TLR4 pathway ultimately leads to the production of pro-inflammatory cytokines, such as TNF [16]. The manner by which Flii alters macrophage TNF secretion was therefore analysed. Mouse peritoneal macrophages obtained from Flii^+/−^, WT, and Flii^Tg/Tg^ mice were stimulated with LPS, and the media was analysed for secreted TNF over a 3-day timecourse. Altering the level of Flii changed both the timing and the amount of TNF secreted, with lower levels of Flii leading to an increased and earlier resolving peak of TNF secretion (Figure 3a). At 3 h post-LPS stimulation, Flii^+/−^ and WT macrophages were secreting TNF, but TNF was not detected in the media of the Flii^Tg/Tg^ macrophages until the 6-hour timepoint, and its level in the media was significantly lower (5-fold less) compared to Flii^+/−^ and WT macrophages at all timepoints (Figure 3a). TNF secretion from the Flii^+/−^ macrophages peaked at 24 h post-LPS stimulation, with levels that were 1.5-fold and 6.8-fold higher than those secreted by WT and Flii^Tg/Tg^ macrophages, respectively (Figure 3a). In contrast, TNF levels in the media from both WT and Flii^Tg/Tg^ macrophages continued to increase over the 3-day timecourse (Figure 3a). Strikingly, significantly less TNF was secreted by the macrophages expressing high levels of Flii (Flii^Tg/Tg^) overall compared to WT and Flii^+/−^ macrophages (Figure 3a). Thus, macrophages expressing low levels of Flii secreted an increased and much earlier peak of TNF, while macrophages expressing high Flii levels displayed delayed and reduced TNF secretion.

Activation of the TLR4 signalling pathway leads to the phosphorylation of the transcription factor NF-κB and transcription of many target genes, including TNF. In vivo, wounds with low levels of Flii (Flii^+/−^) that had fewer macrophages (Figure 2d) showed a significantly lower number of phosphorylated NF-κB^+^ macrophages by day 3, with levels peaking around day 2 post-injury, compared to WT and Flii^Tg/Tg^ wounds, which peaked on day 3 and day 7, respectively (Figure 3b,c). This resulted in a significantly lower number of TNF^+^ macrophages across the 7-day timecourse as compared to WT and Flii^Tg/Tg^ (Figure 3b,c). On day 1, there were 0.6-fold fewer TNF^+^ macrophages in the wounds as compared to those of the WT and Flii^Tg/Tg^ mice, with around 50% of macrophages being positive for TNF. By day 7, there were 1.5-fold and 2-fold more TNF^+^ macrophages in WT and Flii^Tg/Tg^ mice wounds, respectively, compared to Flii^+/−^ wounds (Figure 3b,c). Not all macrophages were TNF^+^ (Figure 3d). On day 2, only 40% of the Flii^+/−^ macrophages within the wound were TNF^+^ as compared to 53% and 61% of macrophages in the WT and Flii^Tg/Tg^ mice’s wounds, respectively (Figure 3d).

### 3.5. Flii Delays the Number and Timing of M2 Macrophages in the Wound

**Figure 4:**
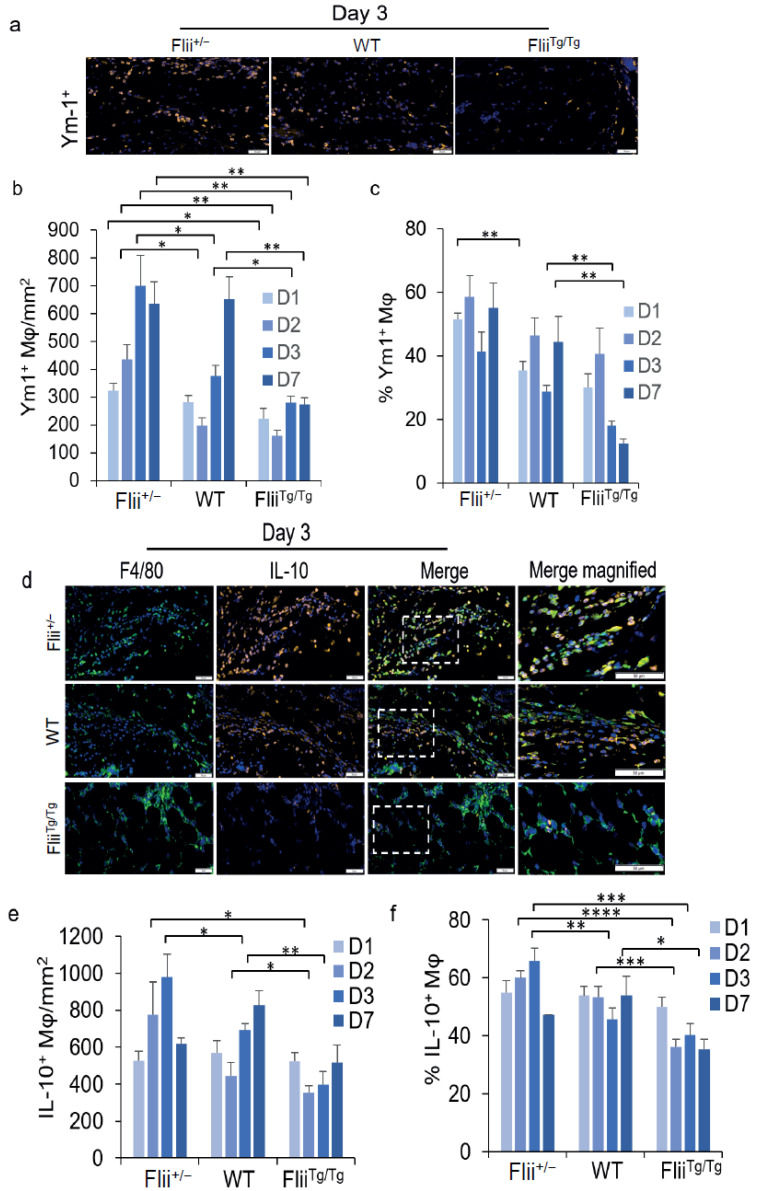
Expression of Ym1 and IL-10 positive macrophages in the wounds from 3 Flii genotypes (Flii^+/^^−^, WT, and Flii^Tg/Tg^): (**a**) Day 3 representative images of wounds immunostained for F4/80 and Ym1, (**b**) the cell counts and (**c**) percentages of Ym-1^+^ F4/80^+^ macrophages present in the wounds of the 3 Flii genotypes (*n* = 6); (**d**) Day 3 representative images of wounds immunostained for F4/80 and IL-10; and (**e**) The cell counts and (**f**) percentages of IL-10^+^ F4/80^+^ macrophages present in the wounds of the 3 Flii genotypes (*n* = 6). Scale bars = 50 µm. Results are expressed as mean ± SEM. * *p* < 0.05, ** *p* < 0.01, *** *p* < 0.001, and **** *p* < 0.0001.

TLR4 signalling is critical for the efficient switching of M1 to M2-like phenotypes [26]. Whether alterations in the percentage of TNF^+^ macrophages seen in the 3 different Flii genotypes (Figure 3) reflected changes in the switch to more M2-like macrophages was next tested. Wounds were assessed for F4/80 and the M2 marker Ym1. A significant increase in the number of Ym1^+^ macrophages was observed in the wounds of the Flii^+/−^ mice as compared to the WT and Flii^Tg/Tg^ mice over a 7-day timecourse (Figure 4a,b). By day 2, the number of Ym1^+^ macrophages had significantly increased in Flii^+/−^ wounds as compared to WT and Flii^Tg/Tg^ wounds (2.2-fold and 2.7-fold, respectively) (Figure 4a,b). By day 3, the levels had increased further and remained elevated on day 7 in the Flii^+/−^ mice’s wounds (Figure 4a,b), with 1.9-fold and 2.5-fold more Ym1^+^ macrophages in the Flii^+/−^ mice’s wounds compared to the WT and Flii^Tg/Tg^ mice’s wounds, respectively (Figure 4a,b). By day 7, Ym1^+^ macrophage numbers were similar in WT and Flii^+/−^ mice wounds, but there were 2.5-fold fewer in the Flii^Tg/Tg^ wounds (Figure 4a,b). This suggests that the switch to a predominantly M2 phenotype starts earlier and may be more pronounced when less Flii is present.

M2 macrophages exert immunosuppressive effects through the secretion of anti-inflammatory cytokines, such as IL-10. Analysis of the Flii^+/−^ mice’s wounds on days 2 and 3 showed that there was a significant increase in IL-10^+^ macrophages compared to those of the WT mice, and an additional increase compared to the Flii^Tg/Tg^ mice’s wounds that overexpressed Flii (D2, 1.8-fold and 2.2-fold; D3, 1.4-fold and 2.5-fold, respectively) (Figure 4c,d). The proportion of macrophages expressing IL-10 was also higher in macrophages that had less Flii, with 60% of the Flii^+/−^ macrophages being IL-10^+^, as compared to 53% and 36% of the macrophages in the WT and Flii^Tg/Tg^ mice’s wounds, respectively, by day 2 (Figure 4e). This suggests that Flii is not only regulating the number of monocytes and macrophages in the wound, but it may also be involved in the timing of the shift to a more M2-like macrophage phenotype.

### 3.6. Flii Delays the Rate of Healing

**Figure 5:**
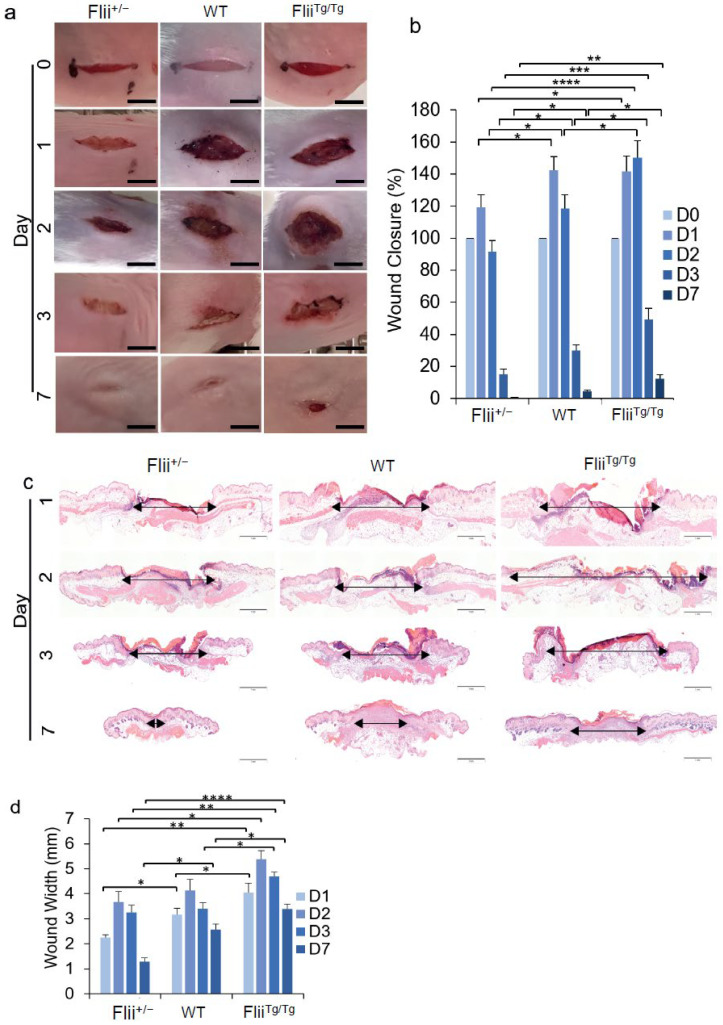
Wound closure in 3 Flii genotypes (Flii^+/^^−^, WT and Flii^Tg/Tg^): (**a**) Representative incisional murine wound photographs from the 3 genotypes over a 7-day timecourse; (**b**) Bar graph showing percentage wound closure (*n* = 6); (**c**) Representative images of H&E staining of the murine incisional wounds; (**d**) Bar graph showing wound width measurements (*n* = 6). Scale bars = 0.5 mm for (**a**), and scale bars = 1 mm for (**c**). Results are expressed as mean ± SEM. * *p* < 0.05, ** *p* < 0.01, *** *p* < 0.001, and **** *p* < 0.0001.

The macroscopic analysis of acute wounds on day 1 showed that Flii^+/−^ mice had a 17% reduction in dermal gape when compared to WT mice; however, there were no significant differences between the WT and Flii^Tg/Tg^ mouse wounds (Figure 5a,b). At day 2, there was a 23% reduction in the size of the Flii^+/−^ wounds and a 21% increase in the Flii^Tg/Tg^ wound dermal gape when compared to the WT wounds (Figure 5a,b). Day 3 Flii^+/−^ wounds had a 50% reduction, and Flii^Tg/Tg^ wounds had a 39% increase in dermal gape when compared to the WT wounds, and at day 7, there were also significant differences, with the Flii^+/−^ wounds showing an 86% reduction and the Flii^Tg/Tg^ wounds showing a 63% increase in dermal gape when compared to the WT wounds (Figure 5a,b). Microscopic analysis showed a 29% reduction in wound width in the Flii^+/−^ wounds, and a 22% increase in the wound width in the Flii^Tg/Tg^ wounds at day 1, when compared to those of the WT (Figure 5c,d). No significant differences were observed at day 2, but at day 3, the Flii^Tg/Tg^ wounds were again 27% larger in wound width compared to the WT controls. At day 7, the Flii^+/−^ wound widths were significantly smaller, and the Flii^Tg/Tg^ wound widths were significantly larger, as compared to the control wounds (49% and 24%, respectively) (Figure 5c,d).

### 3.7. Flii Is Upregulated in the Wounds and Plasma of Diabetic Mice

**Figure 6:**
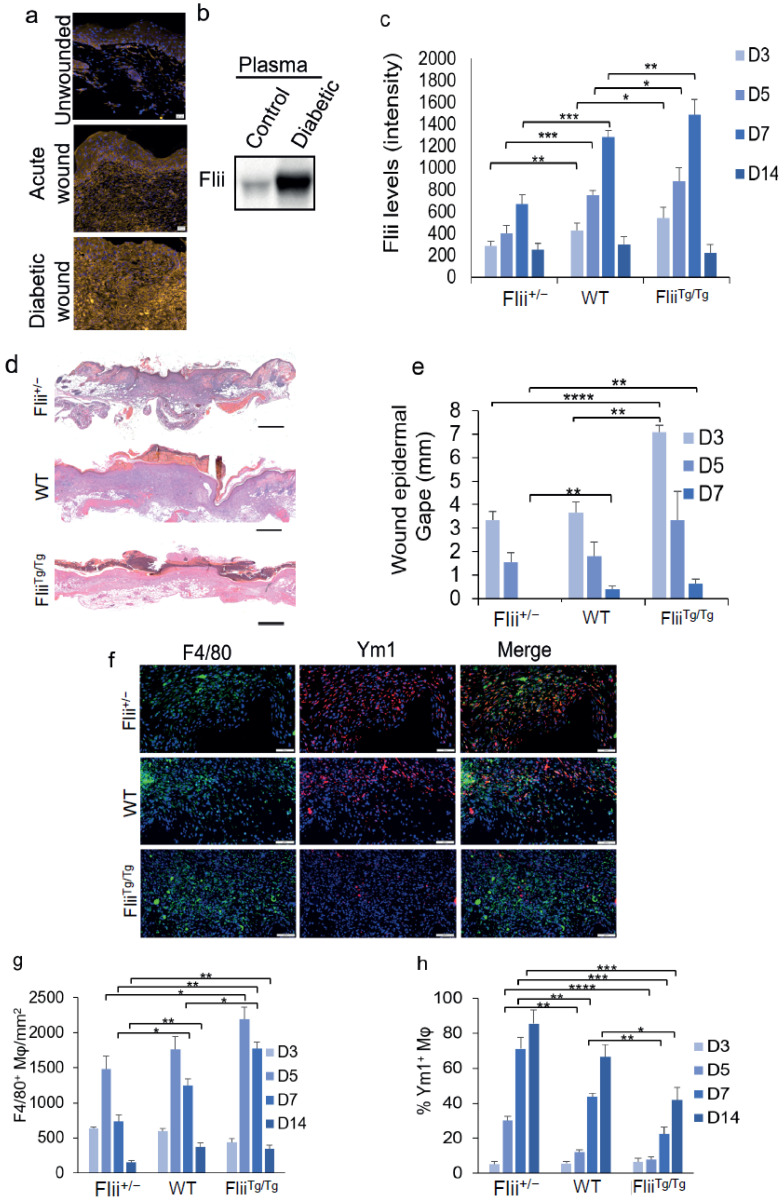
Flii expression and wound closure in a diabetic mouse model: (**a**) Mouse unwounded skin, and acute healthy and diabetic wounds immunostained for Flii; (**b**) Representative Western blot of Flii expression in plasma from diabetic mice; (**c**) Bar graph showing Flii expression levels in murine diabetic wounds in the 3 Flii genotypes; (**d**) Representative H&E staining images of day-7 murine diabetic wounds; and (**e**) Bar graph showing the corresponding wound-gape measurements (*n* = 8); (**f**) Representative images of murine diabetic wounds in the 3 genotypes immunostained for F4/80^+^ and Ym1^+^ on day 7; Corresponding bar charts showing (**g**) the number of F4/80+ macrophages and (**h**) the percentage of Ym1^+^ F4/80 positive macrophages (*n* = 8). Scale bars = 50 µm (**a** and **f**) and 500 µm (**d**). * *p* < 0.05, ** *p* < 0.01, *** *p* < 0.001, and **** *p* < 0.0001.

In uninjured human skin, Flii expression is minimal, and it is significantly upregulated in acute wounds, with a further upregulation in diabetic wounds [14]. Flii is also secreted and can be found in the human plasma of both healthy and diabetic people [14]. To determine whether the same is true for diabetic mice, a murine type-I diabetic model of healing was established using streptozotocin injections and excisional wounds created on the dorsum of each mouse [22]. When compared to unwounded skin and an acute wound in healthy mice, a similar expression pattern was seen; however, unwounded tissue expressed minimal levels of Flii, and levels were increased after injury. These levels were further upregulated in diabetic mouse wounds with impaired healing (Figure 6a). Western blot analysis also showed an increase in the plasma of the diabetic mouse model compared to that of control mice (Figure 6b).

### 3.8. Reduced Levels of Flii Increase M2 Macrophages in Diabetic Wounds and Improve Healing

Diabetes was induced as described above in the 3 mouse genotypes (Flii^+/−^, WT, and Flii^Tg/Tg^), and once the mice were confirmed to be diabetic, excisional wounds were created on the dorsum of each mouse [22]. The expression levels of Flii in the wounds were compared across the 3 genotypes (Figure 6c). Flii^Tg/Tg^ mice expressed the highest level of Flii, followed by WT, with Flii^+/−^ wounds having the least amount of Flii (Figure 6c). The expression of Flii peaked at day 7 in WT and Flii^+/−^ wounds although there was 1.9-fold less in the Flii^+/−^ wounds compared to the WT wounds (Figure 6b). Expression levels in Flii^Tg/Tg^ wounds peaked earlier, at day 5, with 2-fold more than day-5 WT wounds and 2.2-fold more observed in the Flii^+/−^ wounds than that seen in the WT mice on day 3 (Figure 6b). The Flii peak expression in Flii^Tg/Tg^ wounds was 1.2-fold and 2.2-fold higher than the peaks in the WT and Flii^+/−^ wounds. When the wounds were measured macroscopically, it could be seen that the diabetic wounds with lower levels of Flii (Flii^+/−^) had closed by day 7, compared to the WT and Flii^Tg/Tg^ wounds, which had remaining gapes of 0.4 and 0.6 mm, respectively. Interestingly, on day 3, the Flii^Tg/Tg^ wounds had a significantly larger gape of 7 mm, as compared to a gape of 3.6 mm in the WT mice and a gape of 3.4 in the Flii^+/−^ mice.

Staining for F4/80^+^ macrophages showed mice with less Flii (Flii^+/−^) contained fewer macrophages in their wounds, with numbers peaking around day 5 (Figure 6d,e). F4/80^+^ macrophage levels also peaked around day 5 in the Flii^Tg/Tg^ wounds, with 1.2-fold and 1.5-fold more F4/80^+^ macrophages than in the WT and Flii^+/−^ wounds, respectively (Figure 6d,e). Co-staining for F4/80 macrophages with the M2 marker Ym1 showed that the percentages of Ym1^+^ macrophage were low (≤6.5%) on day 3 in all 3 genotypes (Figure 6f). By day 5, the percentage of Ym1^+^ macrophages significantly increased and continued to increase although that increase was significantly lower in mice with more Flii (Figure 6f). In wounds with low levels of Flii (Flii^+/−^), 30% of macrophages were Ym1^+^ on day 5, rising to 85% by day 14; in WT wounds, 12% of macrophages were Ym1^+^ on day 5, rising to 76% by day 14, and in wounds with high levels of Flii (Flii^Tg/Tg^), 8% were Ym1^+^ on day 5, rising to 42% by day 14 (Figure 6F). This suggests that as the level of Flii increases, the shift to the M2-like macrophage phenotype is reduced, which correlates with a delay in healing.

**Figure 7:**
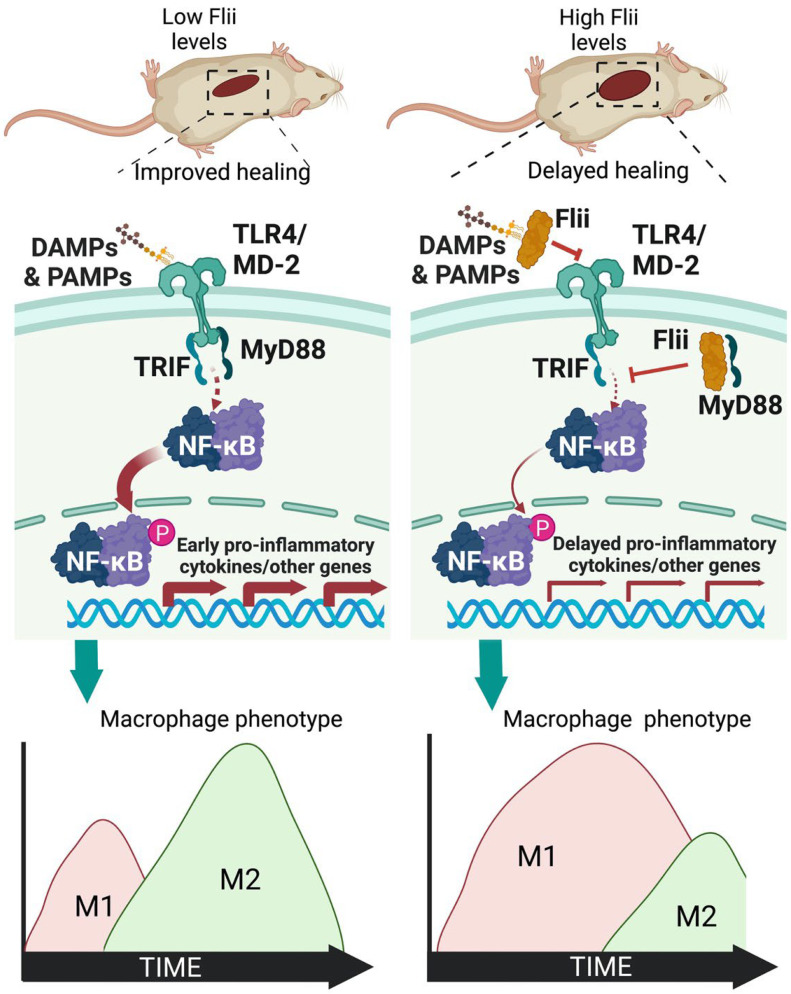
Schematic showing the effect of Flii on TLR4 signalling and wound macrophage phenotypes. As the levels of Flii increase, signalling through the TLR4-NF-κB pathway is dampened, and there is a delay in the shift to M2 reparative macrophage phenotypes in the wound, which delays wound healing. (Created with BioRender.com, accessed on 6 July 2022).

Overall, these data show that high levels of Flii are present in wounds with impaired healing. When Flii levels are high in mice with both acute and diabetic wounds, healing is impaired, and reducing the level of Flii leads to significantly increased wound healing (Figure 7).

## 4. Discussion

Macrophages promote reepithelialisation and wound healing, partly through their ability to alter the local cytokine environment, initially as M1 proinflammatory macrophages and later as more reparative M2 macrophages [27]. Polarisation is facilitated by TLR4 and the downstream outputs of the TLR4 signalling pathway [5,6]. This study shows that increased levels of Flii negatively impact the TLR4 and NF-κB pathways, leading to increased wound inflammation, a delay in the shift from M1 proinflammatory TNF-producing macrophages to more M2 reparative IL-10-producing macrophages, and impaired wound closure. This effect was found to be further exacerbated in diabetic mice which have increased Flii levels prior to wounding. Reducing Flii levels had the opposite effect, with an early shift to M2 macrophages and improved wound closure.

A mounting body of evidence suggests that TLR4-regulated inflammation is important for a timely repair process, specifically TLR4 on the surface of macrophages [2,3,4,6,28,29]. Both Flii and TLR4 are temporally upregulated during wound healing [3,6,12,13]. The data presented here show that Flii negatively regulates the typical increases in surface TLR4 seen after LPS-activation of macrophages, leading to both a delay in timing and a reduction in downstream TNF-secretion in vitro. When Flii levels are low, a shift occurs with an earlier, sharper peak of TLR4-positive cells being observed, as well as increased levels of secreted TNF. TLR4 has also been found to regulate its own transcription after the activation of the TLR4 signalling pathway [30], and we and others have shown that Flii can inhibit TLR4 pathway activation and downstream signalling in vitro [15,16]. This suggests that Flii exerts its effect in a wound, in part, by its ability to finetune TLR4 levels and downstream signalling.

Signalling through the TLR4 pathway leads to alterations in a plethora of genes that regulate many areas of macrophage function. In addition to cytokine secretion, cell migration in several cell types has been shown to be regulated through the TLR4-MyD88/TRIF-NF-κB signalling pathway [31,32,33]. Flii also regulates cell migration [12,13,34]. Fewer infiltrating neutrophils and macrophages are seen in wounds with low Flii levels, which have an early, short peak of cells positive for TLR4 and less inflammation, and the opposite is seen when there is extra Flii present. Whether changes in cytokine secretion, migration per se, or both are responsible for this is currently unclear as both are regulated through TLR4 signalling.

Macrophage polarisation can also be regulated by the TLR4 signalling pathway, with increased levels of TLR4 triggering polarisation towards an M1 proinflammatory phenotype, while TLR4 deficiency promotes M2 polarisation [7]. It has been found in a mouse model of renal injury that a loss of TLR4 not only inhibits macrophage migration, but it also results in a shift to predominantly M2 macrophages at the site of injury [7]. Transition to M2 is linked to improved wound healing. Here, when Flii levels were reduced, an earlier and narrower TLR4 peak was observed, resulting in an increased and earlier peak of M2 wound macrophages, correlating with improved wound closure. The opposite was seen when levels were increased. This suggests that Flii is able to finely tune the TLR4 response and that high levels impede wound repair by negatively impacting TLR4 signalling. Interestingly, the level of Flii, which is typically low in normal skin, was found to be increased in the uninjured skin of diabetic mice and was further upregulated in diabetic wounds. Furthermore, lowering the level of Flii improved diabetic wound closure. These data suggest that blocking Flii would be a reasonable therapeutic strategy for regulating TLR-signalling-pathway-mediated inflammation after injury.

## Data Availability

Data is available upon request to the corresponding author.
